# Supplementary figures and images for: Intestinal Microbiota of Grass Carp Fed Faba Beans: A Comparative Study
Source: Microorganisms. 2019 Oct 17;7(10):465. doi: 10.3390/microorganisms7100465 (PMC6843481; doi:10.3390/microorganisms7100465)

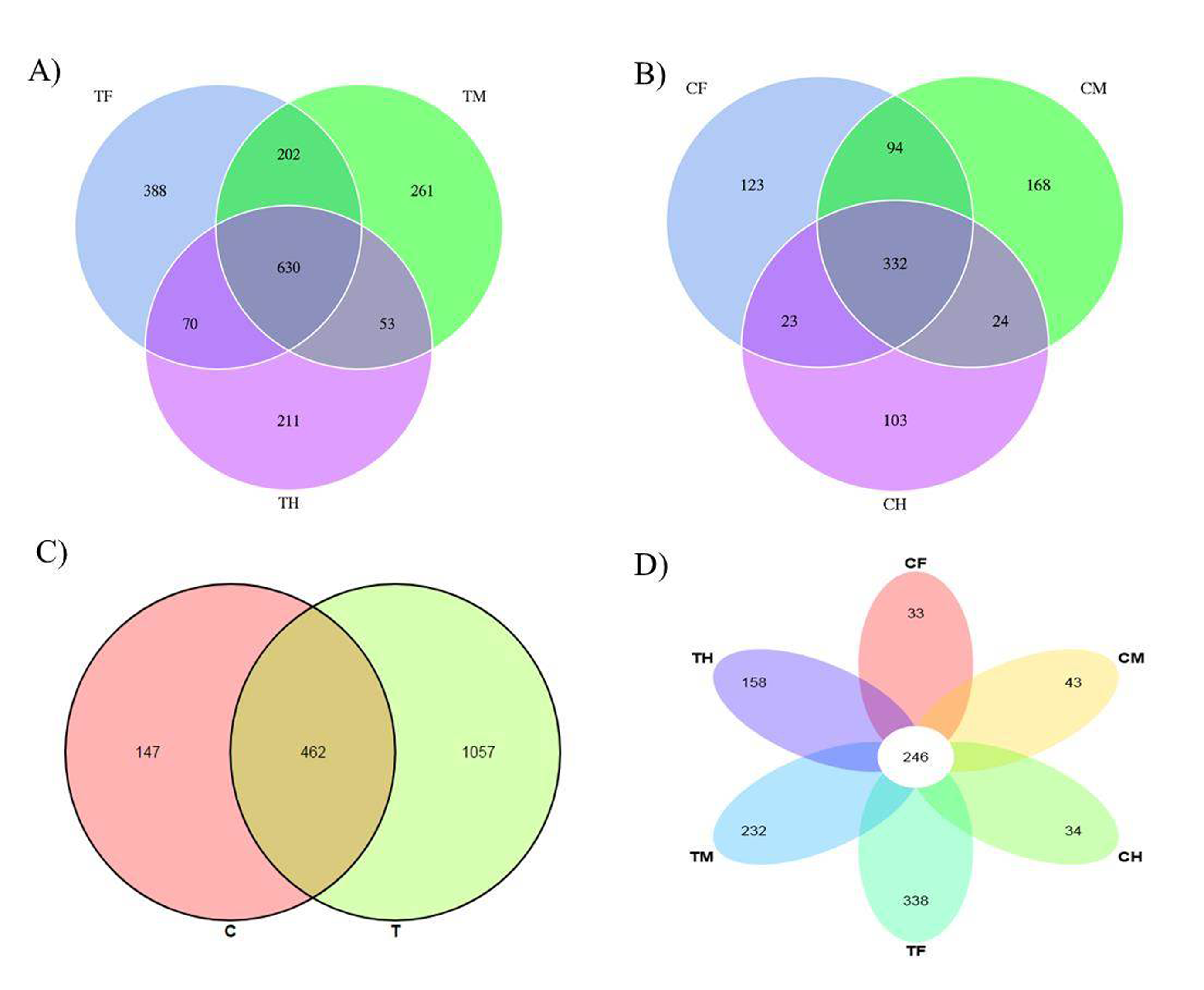

Supplement: Supplementary file 1 [file microorganisms-07-00465-s001.zip › Fig. S1 Venn diagram for intestine microbial OTUs.tif]
